# Congenital Arteriovenous Fistula in a Nine-Year-Old Child: A Case Report

**DOI:** 10.7759/cureus.70645

**Published:** 2024-10-01

**Authors:** Denise Brunozzi, Hamad Farhat

**Affiliations:** 1 Neurosurgery, University of Illinois Chicago, Chicago, USA; 2 Neurosurgery, Advocate Christ Medical Center, Oak Lawn, USA

**Keywords:** carotid-cavernous sinus fistula, congenital arteriovenous fistula, dual microcatheter technique, glue embolization, onyx embolization

## Abstract

Congenital external carotid-jugular fistula is a rare condition caused by altered embryologic development of the vasculature. It is usually treated with surgery or endovascular embolization; controversy exists on the best therapeutic approach and the specific endovascular technique. We report a case of a complex congenital fistula between the external carotid and jugular veins treated with a combined trans-arterial dual microcatheter coiling technique followed by n-BCA (n-butyl cyanoacrylate) glue embolization. After performing a balloon occlusion test to localize the exact fistulous point, dual microcatheter coiling allowed flow reduction and proper packing without balloon assistance. Due to the superficial location of the fistula, the use of n-BCA glue embolization glue guaranteed complete occlusion, avoiding the skin discoloration that Onyx, the standard embolic agent used in fistulas, might cause. This combined endovascular technique provides a safe and simple strategy to optimize efficacy and aesthetic outcomes in a young child with a congenital carotid-jugular fistula.

## Introduction

A congenital external carotid-jugular fistula is a rare vascular developmental anomaly that usually presents in the pediatric population as a pulsatile neck mass or with symptoms and signs of arteriovenous shunting, such as bruit, heart failure, ischemic insult, or ulceration of the skin [1]. Only a limited number of cases have been reported in the literature [1-5]. While the surgical approach was the treatment of choice in the 1970s [2], innovations in the endovascular field have reserved surgery for difficult or failed embolization [6,7] due to high morbidity in cases of venous rupture and potential massive blood loss during the surgical procedure, especially in the pediatric population. Due to the rarity of the disease, there is no consensus on the best endovascular technique for definitive treatment. Here, we present a trans-arterial dual microcatheter coiling technique completed with glue embolization of a complex external carotid artery to internal and external jugular vein fistulas treated at our main institution. We illustrate this case to review the endovascular techniques and literature for the treatment of congenital external carotid-jugular fistulas.

## Case presentation

**Figure 1:**
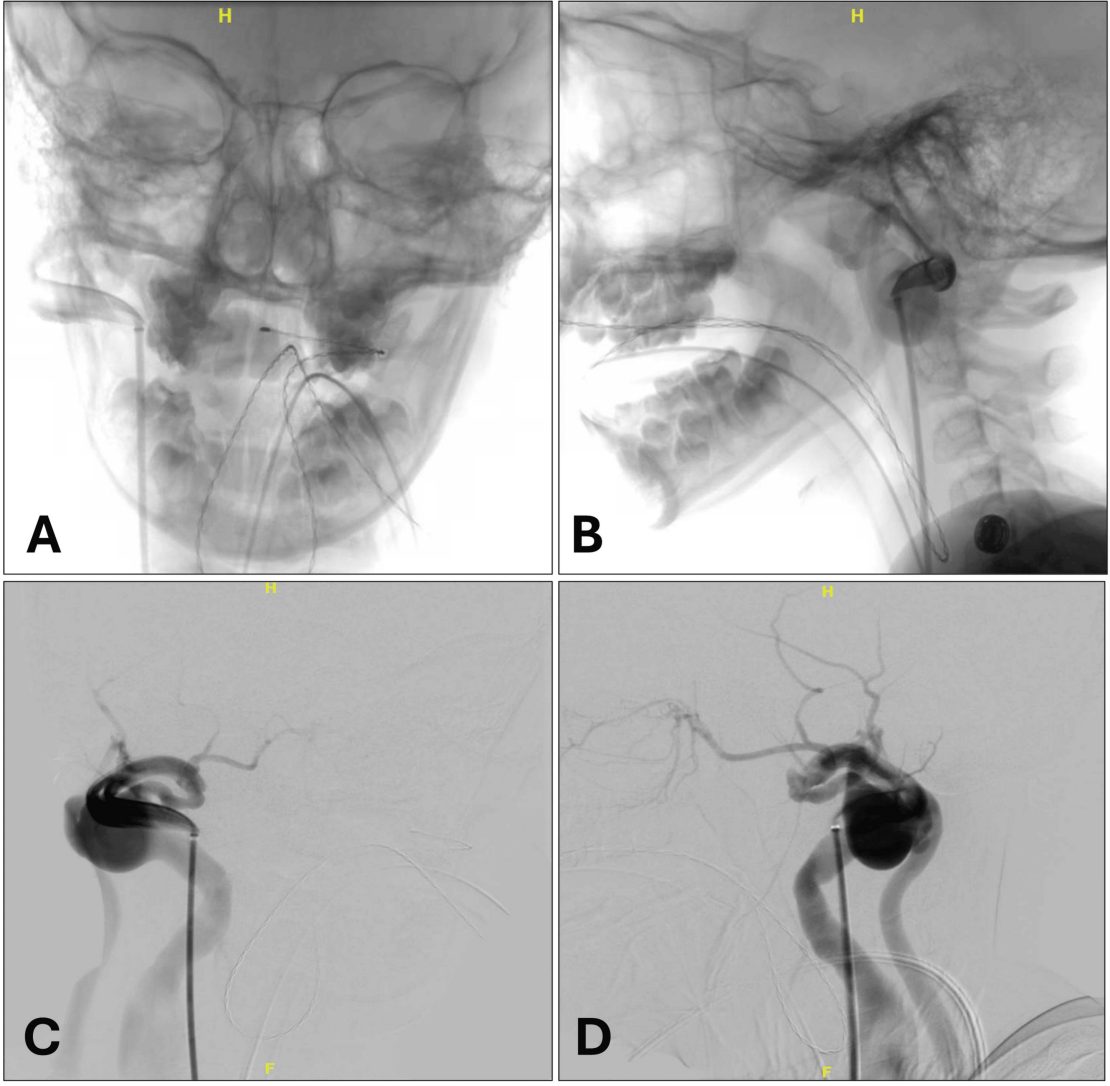
Congenital external carotid-internal and external jugular vein fistula in a nine-year-old child: native anteroposterior (A) and lateral (B) views; respective subtracted anteroposterior (C) and lateral (D) views.

**Figure 2:**
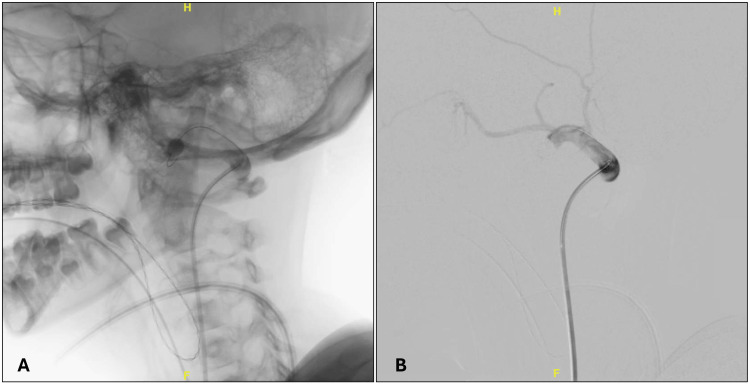
Balloon occlusion test of the fistula, lateral views: native (A) and subtracted (B).

The patient is a nine-year-old female child presenting with a pulsatile neck mass and has a history of a congenital fistula between the external carotid and the internal and external jugular veins, previously treated at our main institution. Due to progressive growth and impact on cardiac function, the decision was made to treat the fistula through an endovascular approach. The complex nature of the fistula and the anatomic location behind the root of the jaw made the surgical ligation not a safe option. Through a 6F femoral artery sheath, a 0.071-inch intermediate catheter was navigated into the external carotid artery proximal to the fistula (Figure 1), and a balloon occlusion test was performed to verify the appropriate location for occlusion and to rule out the presence of additional feeders (Figure 2).

**Figure 3:**
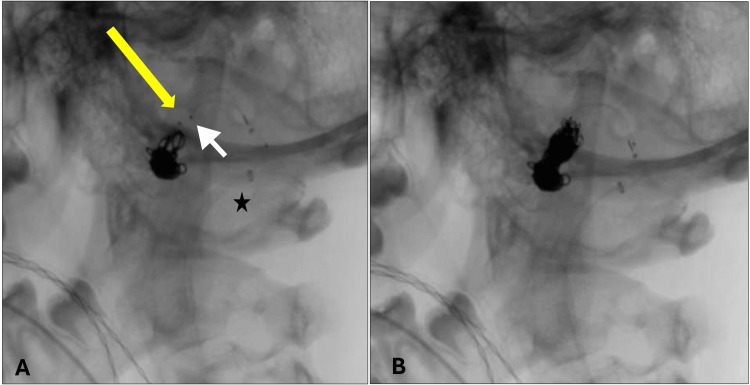
Dual microcatheter coiling technique. (A) The first basket complex coil is deployed through the most distal catheter (yellow arrow: distal marker); the second microcatheter is in place just proximal to the first microcatheter (the white arrowhead indicates the second microcatheter distal marker). The black star indicates the distal guide catheter tip; (B) the soft coil is deployed through the second microcatheter prior to the release of the first coil to weave into each other and create a solid coil mass.

We elected to use a dual microcatheter coiling technique to provide flow reduction with simultaneous multiple coil deployment and efficient packing of the high-flow fistula without the assistance of balloon flow arrest, as previously described in the literature. First, we deployed a complex coil to form a stable mass that slowed the blood flow; then, we used a soft coil to improve mass compaction and create a solid plug (Figure 3).

**Figure 4:**
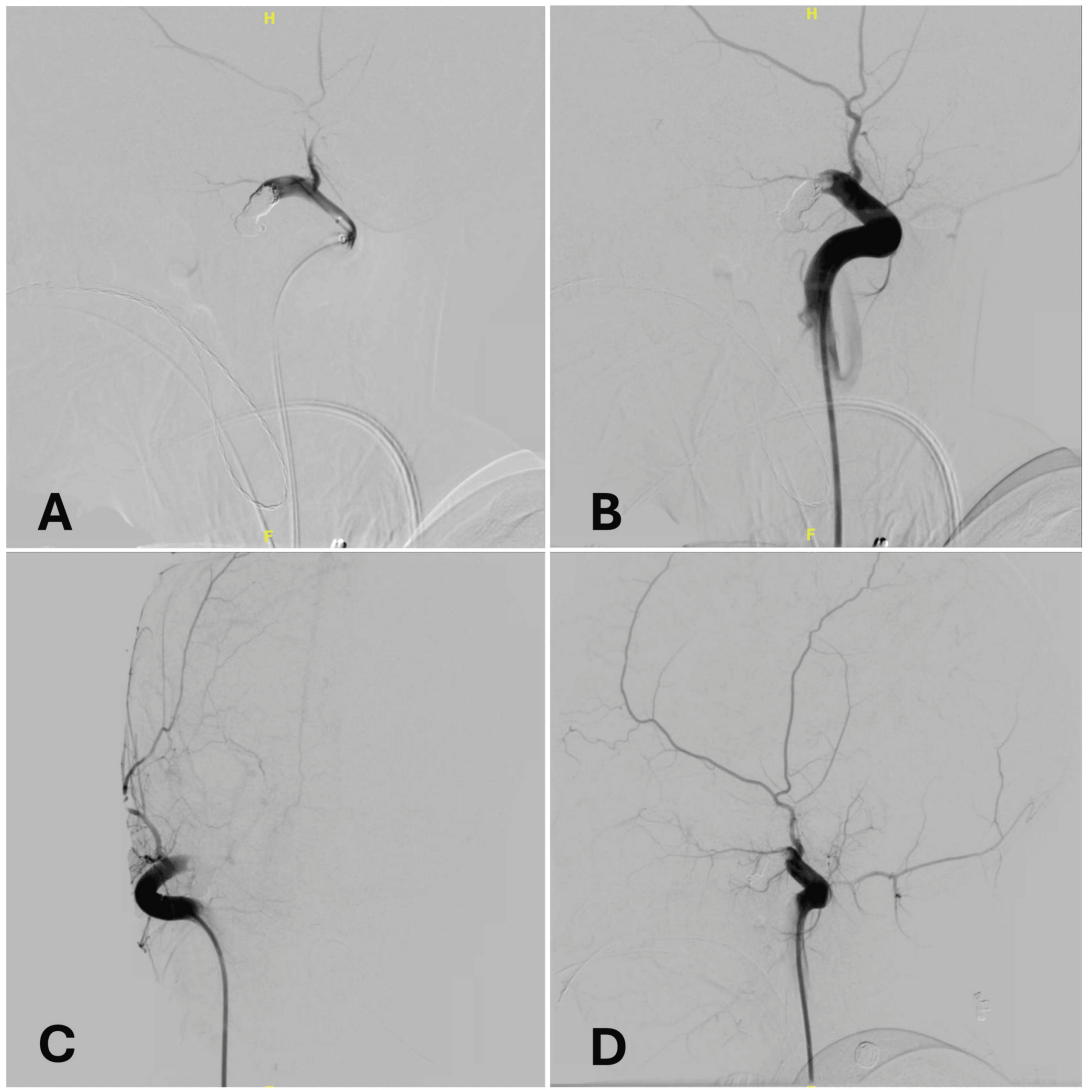
(A) Control angiogram after the completion of coiling (four coils deployed), lateral view; (B) control angiogram after n-BCA glue embolization, lateral view; (C,D) final angiogram with complete obliteration of the carotid-jugular fistula, anteroposterior and lateral views.

Two additional soft coils were used to maximize the packing of the fistula (Figure 4A). To obtain complete obliteration, we added a liquid embolic agent: in this case, due to the superficial location of the fistula and concern for skin discoloration, we chose the transparent n-BCA (n-butyl cyanoacrylate) (Figure 4B), obtaining an optimal result (Figures 4C, 4D). The patient was heparinized during the embolization, and she was reversed with protamine at the end of the procedure.

No complications were encountered; hemostasis at the arteriotomy site was achieved with manual compression, resulting in an immediate near resolution of neck pulsation, and the patient was discharged home the following day.

## Discussion

Congenital arteriovenous fistulas involving the external carotid artery are rare, and only a few cases have been reported in the literature [1-5]. Usually symptomatic, they appear as neck pulsatile masses that tend to grow over time, or they might present with arteriovenous shunting symptoms such as cardiac failure or ischemia. Although the surgical approach is an option, it is usually reserved for complex fistulas with multiple feeders that are not manageable with endovascular treatment or for cases of failed embolization [6,7]. Innovations in endovascular techniques have made embolization the preferable treatment, but no consensus exists on the best endovascular strategy to cure external carotid-jugular fistulas [1,3-5]. These are generally high-flow malformations, and the use of coils or embolization particles to form the plug to close the fistula poses the challenge of increased risk of distal embolization and washout [8]. Closure with a detachable balloon has been advocated as the safest and least traumatic endovascular option to arrest the flow and close the fistula; a few case reports have been described in the literature [3,4,9,10], but long-term results are unknown. Embolization with the use of coils has also been reported in the literature. Bellosta et al. [1] deployed the coils through a balloon guide catheter to decrease the antegrade flow and reduce the risk of distal embolization; Cui et al. [5] instead performed a manual compression on the neck, occluding the external jugular vein during the coiling procedure and completing the occlusion of the fistula with Onyx glue.

Our case report showcases an alternative endovascular trans-arterial approach with coil embolization. The surgical ligation in this case was not considered a safe option due to the complex nature of the fistula and the anatomic location behind the root of the jaw that would have required major invasive surgery and reconstruction.

To ascertain the exact fistulous point where coils needed to be deployed, we initially performed a balloon occlusion test at the fistula to rule out the existence of additional feeders from the external and internal carotid arteries. To overcome the high flow of blood and the risk of distal washout of the coil mass, we adopted the dual microcatheter strategy, previously described for embolization of complex large aneurysms [11,12]. It allows the simultaneous controlled introduction of multiple coils prior to release. In this case, the first coil was deployed through the most distal catheter, which created a basket for the second coil to be deployed through the most proximal microcatheter (Figure 3). Subsequent simultaneous coils were deployed and weaved into each other to form a stable coil mass that prevented coil washout and distal dislodgment.

To optimize the occlusion of the carotid-jugular fistula, we added n-BCA glue to the coil mass. The selection of this embolic agent over the standard Onyx embolization material for fistula treatment was guided by the concern for the cosmetic outcome: being the fistula very superficial, we preferred to use the transparent glue to avoid any evidence of potential skin discoloration that Onyx might have entailed.

To our knowledge, this is the first case of an external carotid-jugular fistula treated with a combined dual microcatheter coiling technique and n-BCA glue embolization. This strategy represents an additional endovascular treatment option for this rare congenital vascular disease.

## Conclusions

A congenital external carotid-jugular fistula is a rare condition that can be treated with a surgical or endovascular approach. Among the endovascular treatment options, coiling carries the risk of washout and distal embolization due to the high flow associated with this lesion. Flow arrest or reduction has been obtained with manual compression or a balloon-assisted guide catheter. Here, we presented a dual microcatheter coiling technique that proved to be a safe and effective method for fistula embolization, overcoming the high-flow-related risk of distal dislodgment with simultaneous controlled deployment of multiple coils prior to release. The closure of the fistula was completed with the use of n-BCA glue, which provided an effective result with an excellent aesthetic outcome. This case illustrates an additional strategy among the several endovascular options available for the treatment of carotid-jugular fistula.
